# Exercise Remodels Akkermansia-Associated Eicosanoid Metabolism to Alleviate Intestinal Senescence: Multi-Omics Insights

**DOI:** 10.3390/microorganisms13061379

**Published:** 2025-06-13

**Authors:** Chunxia Yu, Xuanyu Liu, Yitong Li, Silin Li, Yating Huang, Sujuan Liu, Heng Shao, Yanna Shen, Li Fu

**Affiliations:** 1 School of Medical Technology, Tianjin Medical University, Tianjin 300070, China; yuchunxia@tmu.edu.cn (C.Y.); liuxuanyu0810@tmu.edu.cn (X.L.); lisilin2020@tmu.edu.cn (S.L.); huangyating@tmu.edu.cn (Y.H.); 2 School of Basic Medical Science, Tianjin Medical University, Tianjin 300070, China; liyitong0921@tmu.edu.cn (Y.L.); liusujuan@tmu.edu.cn (S.L.); shaoh@tmu.edu.cn (H.S.)

**Keywords:** exercise, aging, gut microbiota, eicosanoid metabolism

## Abstract

Aerobic exercise mitigates age-related intestinal senescence through gut microbiota modulation, but the underlying mechanism has remained unclear. In this study, we performed 16S rRNA sequencing of gut contents from young, old, and old exercise C57BL/6J mice to assess exercise-induced alterations in microbiota community structure. Differential taxa analyses were applied to reveal age-associated bacterial signatures, gut barrier integrity, and systemic inflammation. Additionally, untargeted metabolomic profiling was employed to characterize gut metabolic profiles and reveal the key pathways through differential metabolite enrichment analyses. Aging significantly exacerbated the senescence-associated secretory phenotypes and the overgrowth of pathogenic bacteria in mice. However, aerobic exercise ameliorated these age-related deteriorations, restored gut microbial homeostasis, and reduced intestinal permeability. Notably, exercise intervention led to a significant increase in Akkermansia abundance in feces, establishing this mucin-degrading bacterium as a prominent exercise-responsive microbe. Metabolomic profiling identified eicosanoid metabolism as the most significantly perturbed pathway, and chronic exercise was found to regulate 14,15-Dhet levels. Our multi-omics integration confirmed that exercise is a potent modulator of the gut–microbiota–metabolite axis during aging. Elucidating the “Akkermansia–eicosanoid signaling” axis provided mechanistic insights into how exercise promotes healthy aging, identifying novel targets for anti-aging strategies via microbiota.

## 1. Introduction

Aging is characterized by progressive deterioration of cellular and tissue function, which significantly elevates vulnerability to various age-associated pathologies, including diabetes mellitus, cardiovascular disorders, and cancer [1]. Reducing the detrimental effects of aging and extending health span have thus been pivotal goals in the fields of aging and anti-aging research. Growing evidence reveals that fundamental aging mechanisms, particularly cellular senescence and chronic low-grade inflammation termed “inflammaging”, are intricately linked with gut homeostasis and microbial imbalance [2]. Alterations in gut microbiota composition have been implicated in exacerbating systemic inflammation and metabolic dysfunction, thereby accelerating physiological decline associated with aging. Elucidating the dynamic interactions between gut microbiota, intestinal integrity, and host metabolism is essential for developing microbiota-targeted strategies to promote healthy aging. This biological framework underscores the intestinal system as a critical hub influencing health outcomes in the aging populations [3,4]. The gut microbiota contributes to systemic homeostasis through multifaceted mechanisms: maintaining gut barrier integrity [5], aiding in nutrient processing [6], modulating the mucosal immune response [7], and preventing pathogen colonization [8]. Notably, age-associated alterations in gut microbiota composition contribute to elevated risks of chronic inflammation and metabolic disorders, potentially through impaired barrier function and immune dysregulation [3]. These findings suggest the gut microbiome as a promising therapeutic target for the development of novel anti-aging therapies.

Exercise, as a non-pharmacological intervention, confers systemic health advantages through intricate molecular mechanisms. Emerging evidence indicates that the modulation of the gut microbiome is a pivotal mediator in the geroprotective effects of exercise [9]. Chronic exercise has revealed its therapeutic potential against metabolic disorders by reshaping microbiota composition, notably increasing the populations of butyrate-producing taxa and reducing pro-inflammatory species [10]. While the age-dependent dysbiosis of gut microbiota is well-documented, the mechanistic interplay between exercise modalities and microbiome aging trajectories remains poorly characterized [11]. Metabolites derived from the gut microbiota are instrumental in sculpting the intestinal microenvironment, regulating systemic organ functions, and preserving immune homeostasis, partly through their dynamic engagements with intestinal epithelial cells [12]. Significantly, aging is accompanied by profound shifts in the host metabolome, which are tightly associated with a range of aging-related diseases [13]. Elucidating the bidirectional linkage between exercise-induced microbial remodeling and metabolomic changes may provide novel insights into microbiota-targeted strategies for fostering healthy aging.

Here, we draw particular attention to address how exercise influences the aging microbiome and associated metabolites. We employed a multi-omics integration strategy, combining 16S rRNA sequencing with comprehensive untargeted metabolomics, to systematically investigate the attenuation of inflammaging through microbiota-dependent pathways in aging mice by exercise. Our approach involved a systematic analysis of gut microbiota and functions using 16S rRNA sequencing, thereby delineating the role of microbial fluctuations in inflammaging and underscoring the protective influence of exercise on age-related changes in gut structure. Furthermore, we explored alterations in the metabolic profile of gut microbiota via extensive untargeted metabolomics, offering a systematic overview of the differential metabolites affected by exercise training in aged mice. Overall, our findings clarified the exercise-induced anti-aging mechanisms from a gut microbiota-centric viewpoint, establishing the foundation for the identification of novel biomarkers and potential therapeutic targets for age-related diseases.

## 2. Materials and Methods

### 2.1. Animals

Mice were housed in a temperature-controlled environment, with 12:12 h light/dark cycle and free access to food and water. Twelve-month-old male C57BL/6 mice were randomly divided into 2 groups: old control group (OC, *n* = 5) and old exercise group (OE, *n* = 7). The OE group underwent 8-month aerobic exercise training on a motor-driven rodent treadmill for 5 days per week (60 min/day) at the intensity of 75% VO_2_max (12 m/min). Six-month-old male mice were assigned to the young controls (YC, *n* = 7).

### 2.2. Intestinal Inflammatory and Cellular Senescence

The mRNA expression levels of senescence-related markers (*Cdkn1a*, *Trp53*, *H2afx*), intestinal permeability markers (*Muc2*, *Ocln*) and pro-inflammatory cytokines (*Il1a*, *Il1b*, *Il6*, *Tnf*, *Cxcl9*) in ileum were quantified using quantitative reverse transcription PCR (qRT-PCR) as described previously. Briefly, frozen ileal tissues stored at −80 °C were homogenized in TRIzol™ reagent (Thermo Fisher Scientific, Waltham, MA, USA) for total RNA extraction. qRT-PCR amplification was conducted using FastStart Universal SYBR Green Master Mix (ROX) (Roche Diagnostics, Basel, Switzerland). Gene expression quantification of *Cdkn1a*, *Trp53*, *H2afx*, *Muc2*, *Ocln*, *Il1a*, *Il1b*, *Il6*, *Tnf*, and *Cxcl9* was performed using the comparative threshold cycle (2−ΔΔCt) method. Actin served as the endogenous reference gene for data normalization. Primer sequences used were listed in Table 1.

### 2.3. Microbiota Analysis

Fecal samples were collected in sterile tubes and immediately frozen in liquid nitrogen. Samples were stored at −80 °C until processing for DNA extraction. The fecal DNA samples were obtained, amplified, quantified, and sequenced using the Illumina MiSeq platform (Illumina, San Diego, CA, USA). Microflora detection was completed by Shanghai Majorbio Bio-pharm Technology Co., Ltd. (Shanghai, China).

### 2.4. Untargeted Metabolomic Analysis

Gas chromatography–mass spectrometry (GC-MS)-based metabolomic analysis was conducted by Majorbio Bio-pharm Technology Co., Ltd. (Shanghai, China). For fecal samples, aqueous homogenates were prepared by mixing samples with deionized water followed by vortex agitation and centrifugation. The resulting supernatant was aliquoted into GC vils containing predetermined internal standards. This mixture underwent solvent evaporation using a controlled nitrogen stream, after which methoxyamine hydrochloride in pyridine solution was introduced. Following vigorous vortex mixing, the solution was maintained at 37 °C for 90 min to complete oximation. Subsequent derivatization was achieved by introducing BSTFA (containing 1% TMCS) to the reaction system. Final derivatized samples were subjected to chromatographic separation using an Agilent 7890A gas chromatograph equipped with a HP-5MS fused-silica capillary column, interfaced with an Agilent 5975C inert MSD mass spectrometer (Agilent Technologies, Santa Clara, CA, USA). High-purity helium served as the carrier gas with optimized constant flow parameters. To minimize analytical bias, sample injection sequences were randomized throughout the analytical process.

Metabolomics data was preprocessed and analyzed through the “MetaboAnalystR” package. First, the relative matrix metabolite was subjected to normalization processing. Subsequently, principal component analysis (PCA) was performed to assess sample distribution patterns. Orthogonal partial least squares discriminant analysis (OPLS-DA) was employed to evaluate the statistical significance of variable importance in the projection (VIP) values for individual metabolites and identify inter-group differential patterns. Differential metabolites were ultimately identified using threshold criteria of VIP ≥ 1 combined with absolute log2-transformed fold-change (Log2FC) ≥ 1.

### 2.5. Statistical Analysis

All experimental data were expressed as mean ± standard deviation (S.D.) of at least three independent experiments. For data analysis, SPSS 22.0 software was applied by two-way ANOVA. To account for multiple comparisons, the Bonferroni method was applied for *p*-value adjustment.

## 3. Results

### 3.1. Exercise Attenuated Age-Associated Intestinal Senescence and Inflammatory Burden in Aged Mice

**Figure 1:**
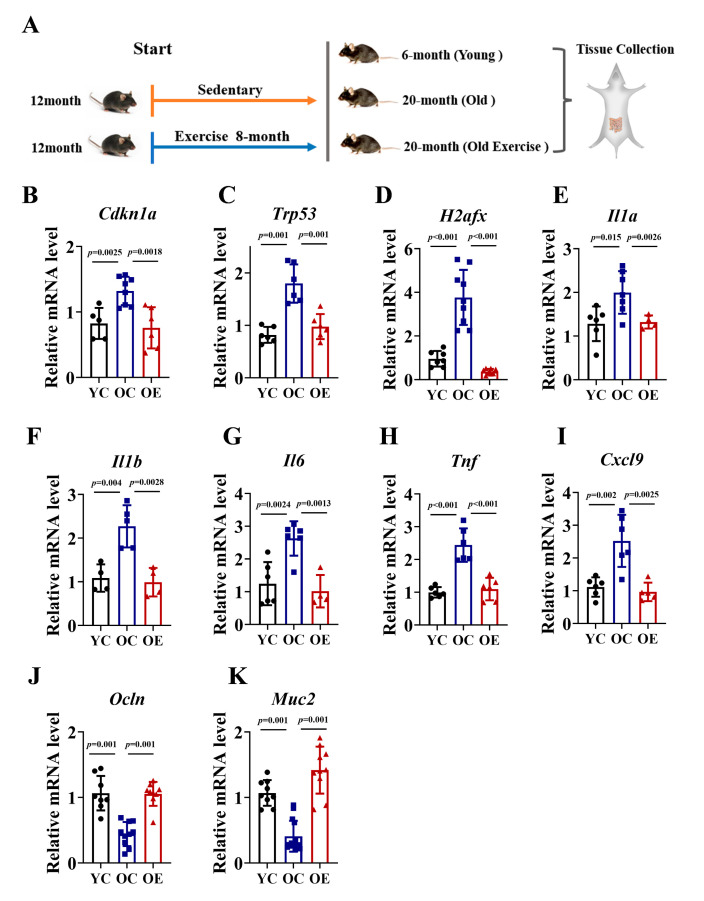
Exercise attenuated intestinal senescence and inflammatory burden in aged mice. (**A**) Schematic of the experimental timeline and exercise regimen. (**B**–**K**) qRT-PCR analysis of senescence markers (*P21*, *Trp53*, *H2afx*) and SASP (*Il1a*, *Il1b*, *Il6*, *Tnf*, *Cxcl9*) in jejunal of YC, OC, and OE groups. Data normalized to YC mice and expressed as mean ± SEM.

To investigate the effects of exercise on intestinal aging, we conducted an 8-month exercise regimen in murine models. We compared ileum tissues from 6-month-old (YC), 20-month-old (OC) mice, and age-matched old exercise (OE) mice (Figure 1A). Given the established role of P21, Trp53 and γH2AX signaling in driving cellular senescence through irreversible cell cycle arrest, we quantified their mRNA expression of these markers as indices of senescence in ileum tissues (Figure 1B–D). In line with the accumulation of senescence during aging, the OC mice exhibited significantly increased mRNA levels of *Cdkn1a* (P21 gene), *Trp53* (Trp53 gene), and *H2afx* (H2AX gene) compared to YC, while the OE mice showed reductions of 43%, 46%, and 24%, respectively. To further characterize senescence-associated secretory phenotype (SASP), we assessed the mRNA expression of pro-inflammatory cytokines, including *Il1a*, *Il1b*, *Il6*, *Tnf* and *Cxcl9*. The OC mice demonstrated a substantial upregulation of all SASP markers compared with the YC mice, indicative of an elevated inflammatory burden. However, chronic exercise effectively attenuated the inflammatory response (Figure 1E–I). The integrity of the intestinal barrier is a pivotal marker of intestinal aging; thus, we quantified the mRNA expression of tight junction proteins (*Muc2*, *Ocln*) across the three groups. Our data demonstrated that the OC mice exhibit a marked reduction in *Muc2* and *Occln* mRNA expression compared to YC mice, whereas chronic exercise significantly reversed this age-associated transcriptional decline. In summary, our findings demonstrated that chronic exercise intervention ameliorated intestinal senescence, reduced inflammatory burden, and preserved gut barrier function in aged mice, underscoring its potential as an effective strategy for combating age-related intestinal deterioration (Figure 1J,K).

### 3.2. Exercise Remodeled Microbiota Composition in Aged Mice

**Figure 2:**
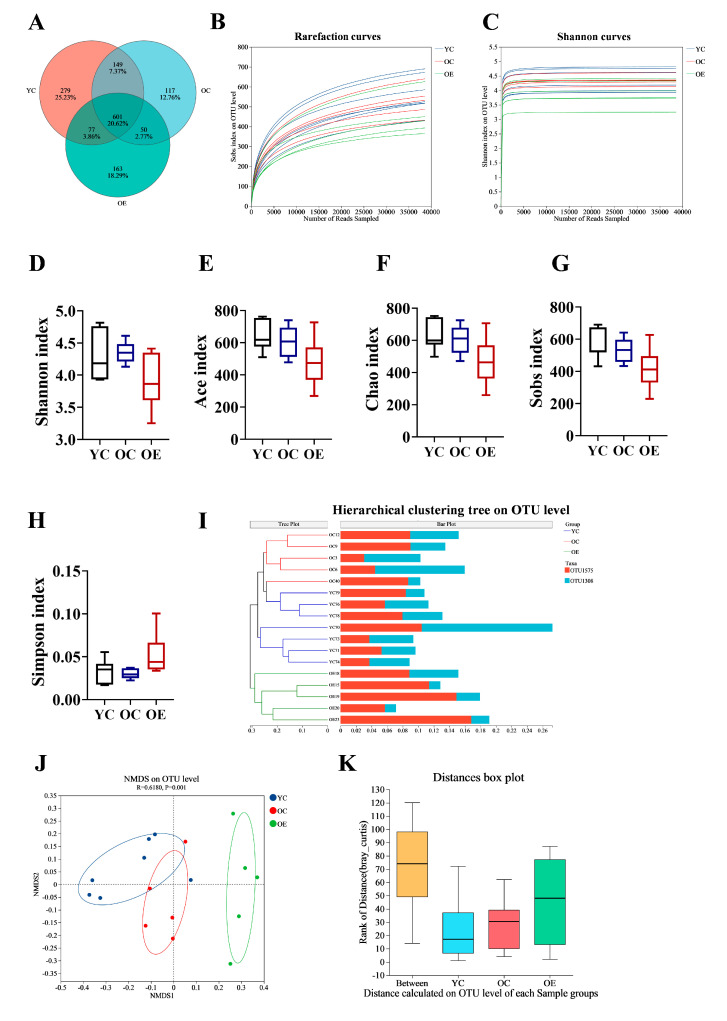
Exercise ameliorated gut microbial dysbiosis in aged mice. Fecal samples were collected from aging mice at the end of study, and 16S rRNA genes in the V3–V4 region were sequenced. (**A**) Venn diagram displayed overlapping and distinct OTUs between groups. (**B**) Rarefaction curve analysis of 16S rRNA gene sequencing depth was performed to assess sample coverage adequacy, with plateau formation indicating sufficient sequencing depth. (**C**) Shannon diversity index rarefaction curves demonstrating sampling sufficiency through asymptotic stabilization patterns. (**D**–**H**) Comparative analysis of alpha diversity indices assessing microbial richness and diversity across different groups (**I**) Hierarchical clustering dendrogram revealing group-specific microbiota compositional patterns. (**J**) NMDS visualization of beta diversity based on Bray–Curtis dissimilarity matrices, demonstrating exercise-induced structural remodeling of gut microbial communities. (**K**) Analysis of similarities (ANOSIM) statistical validation confirming significant inter-group microbiota compositional differences.

Recent findings suggest that the gut microbiota is crucial for preserving intestinal barrier homeostasis and physiological function [14,15]. To investigate the effect of exercise on microbial composition during aging, we performed 16S rRNA sequencing on fecal samples from three groups. We identified a total of 601 operational taxonomic units (OTUs) common to all groups, with 279, 117, and 163 unique OTUs in the YC, OC, and OE groups, respectively (Figure 2A). The rarefaction curves reached a plateau, indicating that the sequencing depth were sufficient to capture the maximum number of species per sample (Figure 2B,C). Alpha diversity analysis (Shannon, ACE, Chao, Sobs, and Simpson indexes) showed no significant differences in microbial richness and diversity across groups (Figure 2D–H). However, beta diversity analysis demonstrated substantial differences in gut microbiota compositions between the groups. Hierarchical clustering and NMDS visualizations based on unweighted and weighted UniFrac distances revealed distinct clustering patterns between young and aged mice (Figure 2I,J). Subsequent adonis analysis using Bray–Curtis distance demonstrated significant differences in microbial composition between groups (*p* = 0.001) (Figure 2K). These results implied that both aging and exercise interventions had a marked impact on the structure of microbial community.

**Figure 3:**
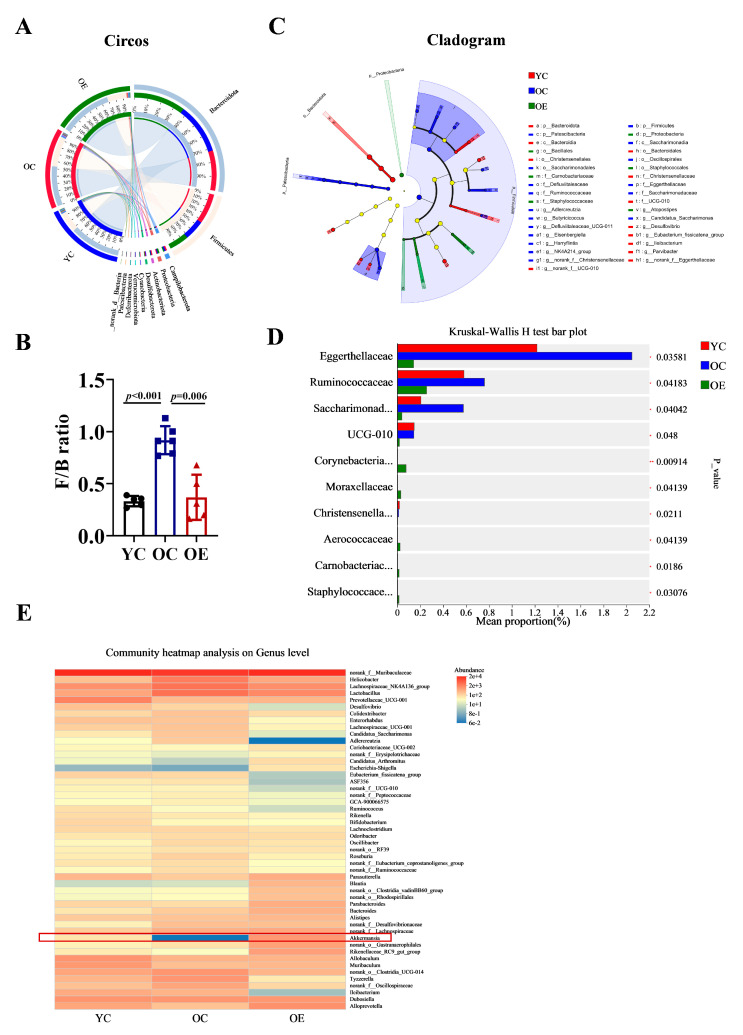
Characterization of discriminative microbial features across three groups. (**A**) Circos analysis displays the corresponding abundance relationship between samples and bacterial communities and (**B**) the F/B ratio was calculated using the Mann–Whitney U test. (**C**) Cladogram showing taxa with a significant difference in relative abundance among the three groups. Nodes colored differently indicate microbial populations that are significantly enriched in their respective groups and demonstrate significant differences among the three groups. The concentric circles from inner to outer represent the taxonomic ranks of phylum, class, order, family, and genus. (**D**) Kruskal–Wallis H analysis of variance bar plot at the phylum level. (**E**) Genus-level microbial profile comparisons through Wilcoxon rank-sum test.

To further characterize microbial shifts at different taxonomic levels (phylum and genus), we performed Circos analysis (Figure 3A). This visualization depicted not only the relative abundances of dominant phyla across the groups but also their distribution patterns. The correlations between samples and microbiota compositions were represented by varying widths of the connecting bands. Bacteroidetes and Firmicutes were identified as the predominant phyla in all groups. The Firmicutes-to-Bacteroidetes ratio (F/B) is a well-established marker of gut microbial composition and metabolic health [16]. Compared with the YC group, the F/B ratio was markedly elevated in the OC group, indicative of age-associated dysbiosis. Interestingly, chronic exercise intervention significantly reduced the F/B ratio from 0.918 to 0.369, thereby restoring microbial balance (Figure 3B). A cladogram depicting the phylogenetic distribution of microbial taxa further revealed age-related microbiota alterations (Figure 3C). The phylum Patescibacteria, known for its pro-inflammatory characteristics [17], was enriched in the OC group. At the family level, aging was associated with an increased abundance of Eggerthellaceae, Ruminococcacea and Saccharimonadaceae. Among them, Eggerthellaceae has been negatively correlated with gut barrier function, while Saccharimonadaceae has been positively associated with inflammatory biomarkers (Figure 3D). Furthermore, we analyzed the distribution characteristics of gut microbiota at the genus level. Notably, the genera Erysipelotrichaceae, Candidatus_Arthromitus, Akkermansia, and Rikenellaceae were significantly reduced in the OC group. Whereas, Helicobacter, Lactobacillus, Colidextribacter, Candidatus_Saccharimonas, Adlercreutzia, and Ruminococcaceae exhibited substantial expansion. Importantly, chronic exercise intervention effectively reversed these age-associated dysbiotic alterations, with particularly pronounced restoration of Akkermansia abundance (indicated by red rectangular), which correlated with improved gut barrier function and enhanced muscle strength (Figure 3E).

### 3.3. Exercise Attenuated Senescence and Inflammation Through Microbiome-Associated Modulation in Aged Mice

**Figure 4:**
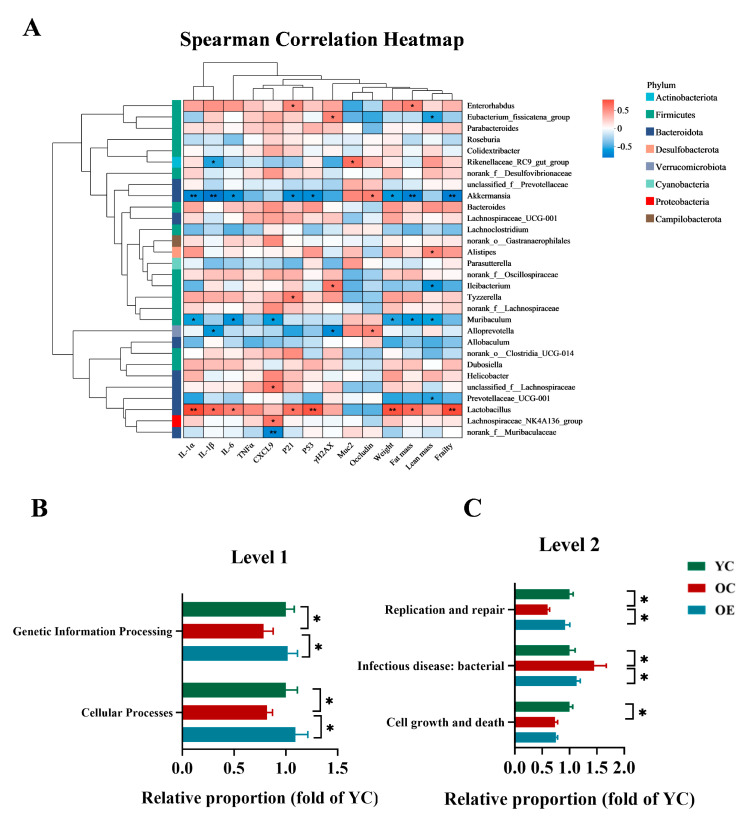
Functional prediction analysis of bacterial microbiota and their biological significance under the aging condition. (**A**) Heatmap visualization of Spearman correlation coefficients between dominant bacterial genera (relative abundance > 1%) and key aging-related metabolic indices. The color gradient denotes correlation strength (red: positive; blue: negative). (**B**,**C**) PICRUSt2 (Phylogenetic Investigation of Communities by Reconstruction of Unobserved States v2.1.4) combined with the KEGG database to predict the function of bacterial microbiota in tissues, showing the results of the KEGG pathway in Level 1 and Level 2. * *p* < 0.05, ** *p* < 0.01.

To elucidate the microbiota–host interactions underlying the pathophysiology of aging, we conducted integrative correlation analyses linking microbial taxa with key hallmarks of senescence, inflammatory mediators, barrier integrity, and frailty indices. Multivariate assessments combined Spearman correlation metrics with hierarchical clustering heatmaps, revealing specific arrays of positive and negative correlation of aging phenotype markers with specific microbiota clades. As shown in Figure 4A, taxa such as Akkermansia, Alloprevotella, Muribaculum, Prevotellaceae_UCG-001, predominantly from the Bacteroidota and Verrucomicrobiota phyla, exhibited inverse correlations with markers of cellular senescence, inflammatory, intestinal permeability, fat mass and frailty. Conversely, Lactobacillus, Lachnospiraceae_UCG-001, Bacteroides, Tyzzerella, Dubosiella, unclassified_f__Lachnospiraceae, Helicobacter, primarily within Firmicutes, demonstrated significant positive correlations with these aging-associated parameters. Notably, Akkermansia emerged as the most potent microbial modulator of aging phenotypes, demonstrating robust inverse correlations with SASP markers and frailty indices, while positively associating with gut barrier restoration. These findings reinforced the critical role of Akkermansia in mitigating age-related deterioration and highlighted its potential as a key microbial target for anti-aging interventions. To further explore the functional implications of microbial remodeling, we performed phylogenetic investigation of communities by reconstruction of unobserved states (PICRUSt) to predict the microbiota-derived functional pathways and assess their biological significance (Figure 4B,C). Functional enrichment analysis revealed that exercise significantly enhanced functions related to genetic information processing and cellular functions at level 1 according to the Kyoto Encyclopedia of Genes and Genomes (KEGG) classification in aging mice. Moreover, level 2 analysis revealed that aging was associated with a marked decline in cellular growth and death, infectious disease resistance, and DNA replication and repair, whereas exercise intervention effectively restored replication and repair-associated pathways. Collectively, these findings indicated that microbial compositional shifts are mechanistically linked to organismal aging, and exercise mitigated dysbiosis-induced replicative stress, contributing to improved cellular resilience and the systemic homeostasis.

### 3.4. Exercise Changed the Microbial Metabolites in Aged Mice

**Figure 5:**
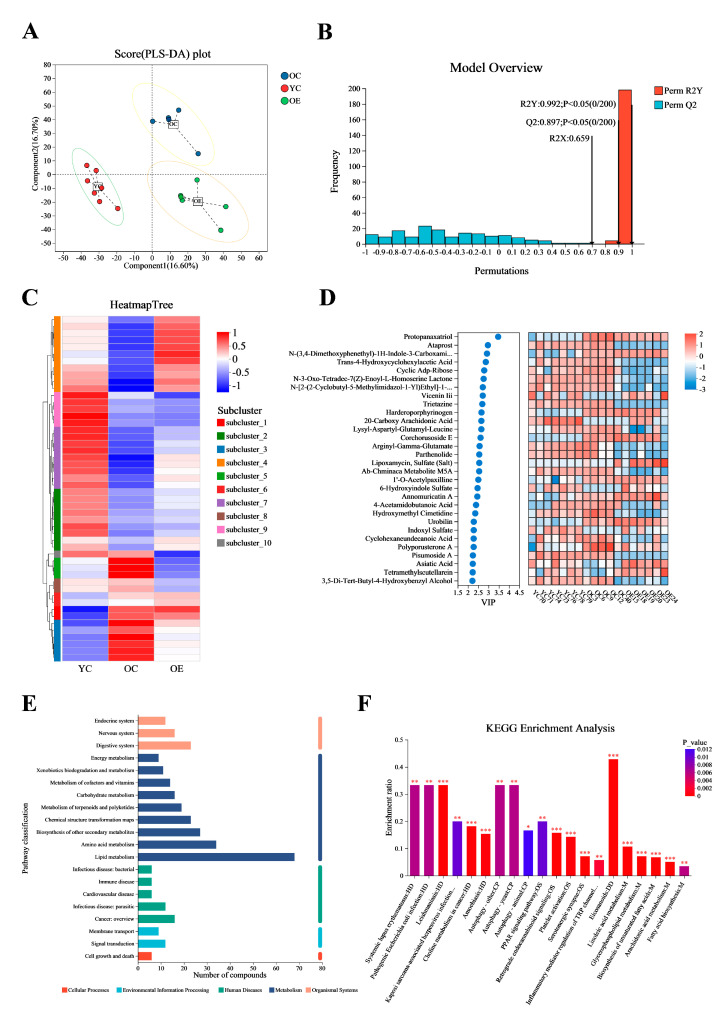
Exercise changed the metabolite contents in fecal matter of aged mice. Ultra-high performance liquid chromatography–tandem mass spectrometry (UHPLC-MS/MS) identified 1061 metabolites. (**A**) OPLS-DA score plot demonstrating distinct metabolic clustering among three groups. (**B**) OPLS-DA model quality parameters and the respective permutation test displaying the statistical validity of the analysis and indicating distinct metabolic profiles in different groups. (**C**) Hierarchical clustering analysis of different metabolites in the mouse fecal. (**D**) Top 30 metabolites ranked by VIP values in the OPLS-DA. (**E**,**F**) Metabolic pathway enrichment of differential metabolites. * *p* < 0.05, ** *p* < 0.01, *** *p* < 0.001.

Microbial metabolites have been recognized for their significant role in influencing and modulating various organ functions [18]. These metabolites may impact host aging and longevity through a variety of mechanisms. In this study, we conducted an untargeted metabolomic profiling of intestinal contents from young and old mice using ultra-performance liquid chromatography–tandem mass spectrometry (UPLC-MS/MS). Figure 5A presented significant differences in intestinal metabolites between groups as determined by partial least squares discriminant analysis (PLS-DA). Subsequent model validation through permutation testing—(*n* = 200), Q^2^ = 0.891, R^2^Y = 0.993, *p* < 0.05—indicated good fitting accuracy of the PLS-DA (Figure 5B). A total of 1061 known metabolites in the cecum content of mice were identified. Heatmaps constructed based on differential metabolite ions among three groups showed a clear clustering (Figure 5C), further confirming the reliability of the PLS-DA models in distinguishing between different metabolic phenotypes associated with aging and exercise. Figure 5D illustrated the top 30 metabolites ranked by their VIP scores in the OPLS-DA analysis. Notably, protopanaxatriol exhibited the highest VIP score across all groups. Subsequently, all 1061 metabolites were classified into 20 KEGG second-grade pathways, and the metabolites were primarily enriched in “lipid metabolism” categories (Figure 5E). To explore the signaling pathways potentially influenced by exercise and aging metabolites, we performed KEGG pathway enrichment analysis. The results indicated that “eicosanoids” may be the most significantly affected signaling pathways during exercise process (Figure 5F).

### 3.5. Exercise Mitigated Intestinal Aging Through Eicosanoids Pathways

**Figure 6:**
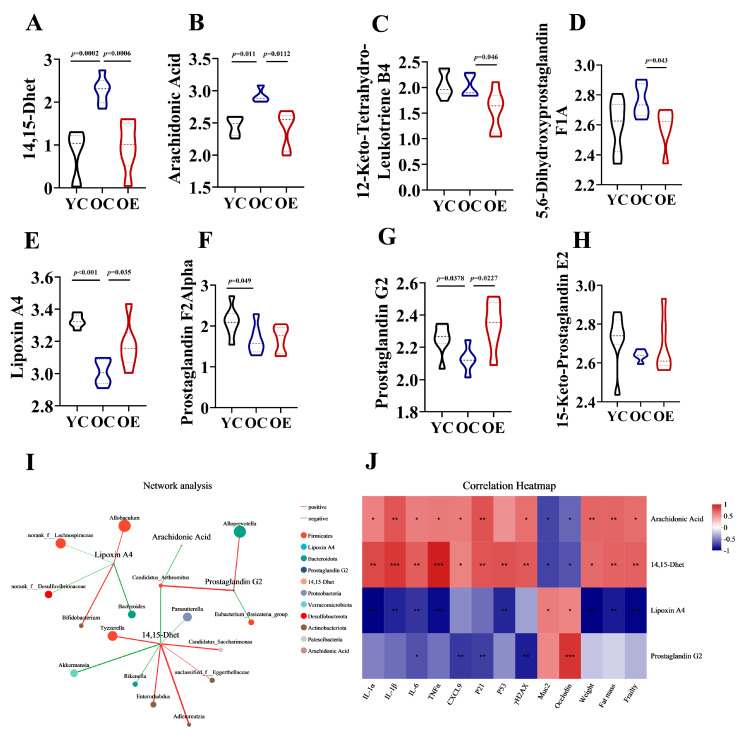
Exercise-attenuated-aging phenotypes by regulation of eicosanoid metabolism. (**A**–**H**) Quantification of eicosanoid metabolites in the feces of mice. (**I**) Network analysis among different intestinal microbiota and eicosanoid metabolites based on Spearman’s analysis. (**J**) Association of eicosanoid metabolites with vital aging metabolic parameters. * *p* < 0.05, ** *p* < 0.01, *** *p* < 0.001.

Eicosanoids are lipid signaling molecules derived from the oxidation of ω-6 fatty acids [19], which play critical roles in inflammatory regulation. Our analysis revealed that aging and exercise significantly influenced eicosanoid metabolism. We measured the levels of eight key metabolites linked to this pathway in fecal samples, including 14,15-Dhet, arachidonic Acid, 12-Keto-Tetrahydro-Leukotriene B4, 5,6-Dihydroxyprostaglandin F1A, lipoxin A4, prostaglandin F2Alpha, prostaglandin G2 and 15-Keto-Prostaglandin E2 in mouse feces (Figure 6A–H). In OC mice, we observed increased intestinal levels of pro-inflammatory metabolites 14,15-Dhet and arachidonic acid, alongside reduced levels of the anti-inflammatory mediators lipoxin A4 and prostaglandin G2 compared to YC mice. Exercise intervention normalized these age-related imbalances, suppressing lipoxin A4 and arachidonic acid accumulation. Notably, dysregulation of eicosanoid networks previously implicated in chronic inflammation [20], hepatic pathophysiology, and carcinogenesis, suggests that eicosanoid disturbances may contribute to not only altered lipid metabolism but also be associated with aging. After characterizing the metabolites in the intestinal contents of aged and exercise groups, we further explored their correlations with aging-associated differential genera through Spearman correlation analysis. Network analysis visualized the following associations: Node color corresponds to phylum taxonomic classification. Edge color represents positive (red) and negative (green) correlations, and the edge thickness is equivalent to the correlation values. As shown in Figure 6I, most genera from the Firmicutes phylum exhibited positively with 14,15-Dhet and negatively with Lipoxin A4 and Prostaglandin G2. Conversely, Alloprevotella demonstrated a positive correlation with Prostaglandin G2, and Akkermansia showed negatively correlated with 14,15-Dhet. Correlation heatmaps further uncovered the roles of eicosanoid metabolites in gut aging. 14,15-Dhet and arachidonic acid correlated positively with senescence markers (*P21*, *Trp53*, *H2afx*), SASP factors (*Il1a*, *Il1b*, *Il6*, *Tnf*, *Cxcl9*), fat mass, and frailty, while showing an inverse correlation with barrier integrity. Conversely, Lipoxin A4 and Prostaglandin G2 exhibited diametrically opposed associations. Notably, 14,15-Dhet exhibited the strongest associations with cellular senescence and microbiota composition (Figure 6J). These data delineated an Akkermansia-driven lipid signaling axis, wherein microbial dysbiosis amplifies production of 14,15-Dhet, thereby exacerbating inflammaging and cellular senescence. Exercise-mediated microbiota restoration rebalanced eicosanoids pathways, suppressing pro-aging metabolites (14,15-Dhet and arachidonic acid) while enhancing barrier-protective Lipoxin A4 and Prostaglandin G2.

## 4. Discussion

Accumulating evidence suggests that gut dysbiosis is closely associated with the pathophysiology of aging, yet the precise mechanisms remain elusive [21,22]. The intestine is involved in many physiological processes, encompassing nutrient absorption, immune modulation, and interaction with the commensal microbiome [23]. It presents an important physical and chemical barrier to the external environment while also functioning as an integration site that responds to various physiological and pathophysiological stimuli, including microbial metabolites, dysbiosis and inflammation-associated processes [24,25]. Emerging evidence suggests that unhealthy perturbations in gut microbiota are related to disrupted host nutrient signaling pathways and metabolism [25], which may contribute to the development of age-related diseases and adversely affect the overall health of the host [26].

Habitual exercise has been consistently validated as an effective non-pharmacological strategy to enhance health across the lifespan [27]. Exercise has been shown to positively change microbiota composition and diversity, and exercise-induced gut microbiota reshaping independently of dietary patterns [10,15]. In the present study, we observed that the OE mice, in addition to exhibiting distinct microbiota signatures, also exhibited reduced intestinal senescence and inflammation burden compared to the OC group. Beta diversity analysis further confirmed that aging was accompanied by shifts in microbial communities. In particular, we identified an enrichment of harmful and opportunistic pathogens in the aged group. Notably, the phyla Firmicutes and Patescibacteria were found to possess potent pro-inflammatory capabilities [17] and the family Eggerthellaceae has been demonstrated to be correlated with gut barrier dysfunction [28]. Chronic exercise significantly enhanced the relative abundance of beneficial gut microbiota in the OE group, especially the Akkermansia species, which were significantly inversely correlated with key senescence-associated phenotypes, including intestinal permeability, inflammatory markers, and epigenetic aging parameters [29]. These findings underscored the critical role of exercise in remodeling the gut microbiome and mitigating the effects of aging by fostering a healthier, and more balanced microbial ecosystem.

Within the mucosal layer, microbial communities actively influence host physiology through the secretion of metabolites or by-products derived from bacterial turnover, which contributes to the maintenance of local intestinal homeostasis [30]. Aging is known to induce alterations in the metabolic profile, which may serve not only as potential markers of aging process but also as factors that could reverse aging phenotypes or treating age-related diseases [31,32,33]. However, the metabolic alterations associated with the aging process are not yet fully understood. Herein, untargeted metabolomics profiling demonstrated a predominant enrichment of differential metabolites within lipid metabolism networks, particularly in eicosanoids pathways. We observed notable discrepancies in the abundance of various eicosanoid metabolites, including 14,15-Dhet, arachidonic acid, lipoxin A4 and prostaglandin G2. Of note, 14,15-Dhet, an omega-6 oxylipins, has been identified as an endogenous signal of inflammation [34]. Previous studies have reported that 14,15-Dhet impairs neutrophil chemotaxis, acidification, CXCR1/CXCR2 expression, reactive oxygen species (ROS) production, and exacerbated systemic inflammation [35]. While the role of 14,15-Dhet in specific diseases has been described, its function in aging tissues or organs remains largely unexplored. Our results revealed that 14,15-Dhet acted as a pro-inflammatory lipid mediator, exhibiting strong inverse correlations with Akkermansia abundance and positive associations with SASP markers. Exercise-induced Akkermansia enrichment was associated with reduced 14,15-Dhet accumulation, concomitant with a reduction in SASP markers. These findings highlighted the Akkermansia/14,15-Dhet axis as a pivotal microbiota-lipid signaling pathway through which exercise may mitigate intestinal inflammaging.

## 5. Conclusions

Our findings suggested that dysregulated eicosanoid metabolism might serve as a pivotal biomarker of the aging processes and underscore the gut microbiota–eicosanoid axis as a potential therapeutic target for mitigating age-related diseases. Chronic exercise induced remodeling of the gut microbiota composition, characterized by a decrease in the F/B ratio and an increase in Akkermansia abundance, which collectively suppressed 14,15-Dhet accumulation and contributed to the resolution of SASP markers. The “Akkermansia—eicosanoids metabolism-14,15-Dhet pathway” might act as a promising mechanism linking gut microbiota dynamics to aging. These results established a novel connection between exercise-induced microbial remodeling and aging, providing valuable insights for the development of microbiota-targeted therapeutic strategies aimed at mitigating age-related pathologies. This study established a valuable foundation for preclinical studies to understand and validate the therapeutic potential of exercise in age-related disorders involving senescence, chronic inflammation, and microbiota dysbiosis.

## Figures and Tables

**Table 1:** Primers used in this study.

Gene	Forward (5′-3′)	Reverse (5′-3′)
*Cdkn1a*	GTCAGGCTGGTCTGCCTCCG	CGGTCCCGTGGACAGTGAGCAG
*Trp53*	CCGACCTATCCTTACCATCATCA	AGGCACAAACACGAACCTCAA
*H2afx*	GGCCTGTGGACAAGAGTTCTAT	GCCCATTAAATCTCCCCACT
*Muc2*	GCTGACGAGTGGTTGGTGAATG	GATGAGGTGGCAGACAGGAGAC
*Ocln*	CATCAGCCATGTCCGTGAGG	GGGGCGACGTCCATTTGTAG
*Il1a*	GCACCTTACACCTACCAGAGT	AAACTTCTGCCTGACGAGCTT
*Il1b*	TTGAAGTTGACGGACCCCA	ATGAGTGATACTGCCTGCCTGA
*Tnf*	AGGGTCTGGGCCATAGAACT	CAGCCTCTTCTCATTCCTGC
*Cxcl9*	AGTGTGGAGTTCGAGGAACCCT	TGCAGGAGCATCGTGCATT
*Il6*	CCAGAAACCGCTATGAAGTTCC	TTGTCACCAGCATCAGTCCC

## Data Availability

Data supporting the findings of this study are available at Sequence Read Archive (SRA) database [https://submit.ncbi.nlm.nih.gov/subs/] (Accession Number: PRJNA1263684) and https://ngdc.cncb.ac.cn/omix/preview/LUzkGZdX (BioProject: PRJCA040310).
